# Block copolymer crystalsomes with an ultrathin shell to extend blood circulation time

**DOI:** 10.1038/s41467-018-05396-x

**Published:** 2018-08-01

**Authors:** Hao Qi, Hao Zhou, Qiyun Tang, Jee Young Lee, Zhiyuan Fan, Seyong Kim, Mark C. Staub, Tian Zhou, Shan Mei, Lin Han, Darrin J. Pochan, Hao Cheng, Wenbing Hu, Christopher Y. Li

**Affiliations:** 1 Department of Materials Science and Engineering, Drexel University, Philadelphia, PA 19104 USA; 2 Institut für Theoretische Physik, Universität Göttingen, Friedrich-Hund-Platz 1, 37077 Göttingen, Germany; 3 Department of Materials Science and Engineering, University of Delaware, Newark, DE 19716 USA; 4 School of Biomedical Engineering, Science & Health Systems, Drexel University, Philadelphia, PA 19104 USA; 5 Department of Polymer Science and Engineering, State Key Lab of Coordination Chemistry, School of Chemistry and Chemical Engineering, Nanjing University, 210023 Nanjing, China

## Abstract

In water, amphiphilic block copolymers (BCPs) can self-assemble into various micelle structures depicting curved liquid/liquid interface. Crystallization, which is incommensurate with this curved space, often leads to defect accumulation and renders the structures leaky, undermining their potential biomedical applications. Herein we report using an emulsion-solution crystallization method to control the crystallization of an amphiphilic BCP, poly (l-lactide acid)-*b*-poly (ethylene glycol) (PLLA-*b*-PEG), at curved liquid/liquid interface. The resultant BCP crystalsomes (BCCs) structurally mimic the classical polymersomes and liposomes yet mechanically are more robust thanks to the single crystal-like crystalline PLLA shell. In blood circulation and biodistribution experiments, fluorophore-loaded BCCs show a 24 h circulation half-life and a 8% particle retention in the blood even at 96 h post injection. We further demonstrate that this good performance can be attributed to controlled polymer crystallization and the unique BCC nanostructure.

## Abstract (web summary)

In block copolymer vesicles, crystallization often leads to defects and renders the structures leaky that undermines their potential biomedical application. Here the authors use an emulsion solution method to control the crystallization of an amphiphilic block copolymer at the curved liquid/liquid interface to improve the blood circulation time.

## Introduction

Over the past few decades, many elegant delivery systems emerged to mimic biological structures such as lipid membranes and virus capsid^1–3^. For example, red blood cells (RBCs)-mimicking particles were reported to resemble natural RBCs’ size, shape, and deformability^4–6^. Self-assembled liposomes that mimic envelope viral structures were developed as tumor-targeting gene delivery carriers^7,8^. Like lipids, synthetic amphiphilic block copolymers (BCPs) can also self-assemble into similar vesicle structures (defined as polymersomes to emphasize its synthetic polymer origin) in water^9,10^. Because of the high molecular weight and chain entanglement of the hydrophobic blocks, polymer vesicles are mechanically more stable than liposomes^10^. Yet polymersomes’ membranes are flexible, and their morphologies/shapes may change over time, particularly under a high shear field. While shape transformation of polymersomes is of great physical and biological interest, in vivo applications call for robust and mechanically stable carrier structures.

Strategies towards permanent BCP assemblies include using crosslinking agents to form shell-cross-linked knedel-like (SCK) particles and incorporating BCP with self-crosslinkable segment^11,12^. Early studies in polymersome systems showed that polymer crystallization affected the formation of polymersomes as well as their mechanical properties^13,14^. Discher et al. demonstrated that, after polymer crystallization, vesicles of semicrystalline poly(ethylene oxide)-*b*-polycaprolactone (PEO-*b*-PCL) are rigid and leaky, which was attributed to the defects formed between the adjacent polymer crystalline domains (grain boundaries)^15^. Since many biometrically relevant amphiphilic BCPs are crystallizable at ambient conditions, it is of importance to investigate polymer crystallization at curved nanospace. From a crystallographic standpoint, curved space is incommensurate with three-dimensional translational symmetry. Recent studies on spherical crystallography showed intriguing packing behavior of colloidal particle at the surface of microscale water droplets^16–18^. Intriguing defects or voids were introduced to these curved crystals depending on the nature of particle−particle interaction as well as the size of the particles and water droplets^16–18^. We recently developed a miniemulsion-solution crystallization method to study confined crystallization at nanometer-sized, curved liquid/liquid interface. Polymer-single crystal-like poly (l-lactide) (PLLA) hollow capsules were formed by carefully directing polymer crystallization at curved liquid/liquid interface^19^. *Crystalsome* was used to describe this unique assembly, emphasizing its crystalline structure and vesicle-like morphology. While structurally interesting, these PLLA crystalsomes are hydrophobic and cannot be dispersed in water without added surfactants, limiting their potential biomedical applications.

Herein, we report using the emulsion-solution crystallization method to assemble amphiphilic BCP crystalsomes (BCCs). During the crystallization process, the BCP acts both as the surfactant to stabilize oil droplet in water, and as the crystallizable material to form the crystalsome so that the crystallization process is precisely confined at the curved liquid/liquid interface. Nano-sized BCCs are formed with an asymmetric wall comprised of concentric 2.5 nm PLLA lamellar crystal and a 2 nm PEG layer. In the crystal, each PLLA chain folds nine times, rendering a uniform PEG brush layer with a precisely controlled grafting density of 0.3 chain nm^−2^. While the total wall thickness is only 4.5 ± 0.4 nm (mean ± s.d., *n* = 10), these BCCs show ultra-long circulation time in vivo. We attribute this superb blood circulation performance to the combination of significantly enhanced mechanical properties of the single crystal-like shell and the uniform PEG brush layer. We envisage BCCs lead to a new class of self-assembled nanoparticle delivery systems.

## Results

### Morphology and structure of PLLA-*b*-PEG BCCs

**Fig. 1:**
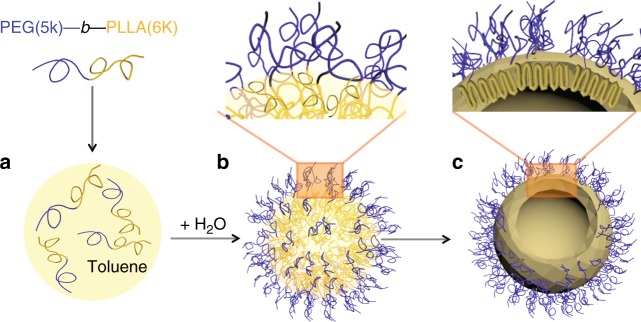
Fabrication of PLLA-*b*-PEG block copolymer crystalsomes. **a** Dissolution of the BCP in toluene; **b** emulsification at 95 °C; **c** quenching to 25 °C for crystallization. The driving force of this assembly process is confined PLLA crystallization at the toluene/water interface, leading to a ninefold PLLA chain conformation as shown in **c**. This confined crystallization process also leads to a 2.5 nm thick PLLA crystal layer, covered with a precisely controlled, uniform PEG brush layer (grafting density of 0.3 chain nm^−2^)

In this study, we selected crystalline and biocompatible polymer PLLA as the hydrophobic segment. PEG is chosen as the second block of the BCP as it affords water solubility and stealthability to increase the particle circulation time in the blood^20^. The fabrication process of PLLA-*b*-PEG block copolymer crystalsome is shown in Fig. 1. In brief, PLLA-*b*-PEG is firstly dissolved in toluene at 95 °C. Water is added into the polymer solution and the mixture is ultrasonicated to generate an emulsion, within which the PLLA segments are confined in the toluene droplet while the PEG segments in the water phase. The emulsion is then quenched to 25 °C for crystallization. In this design, PLLA-*b*-PEG molecules are pinned at the curved liquid/liquid interface after emulsification, crystallization of PLLA is therefore confined at the interface and the formation of more complex spherulites/dendrite crystals inside the droplet is avoided^21^.

**Fig. 2:**
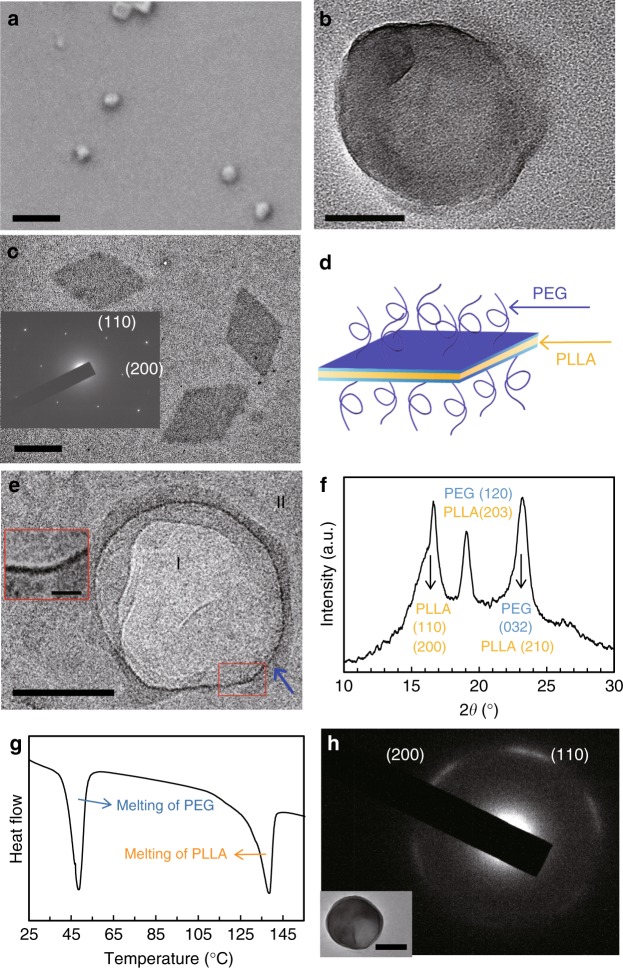
Morphology and crystal structure of PLLA-*b*-PEG single crystal and BCCs. **a** SEM and **b** TEM micrographs of BCCs in dried state. **c** TEM micrograph of flat PLLA-*b*-PEG polymer single crystals grown from toluene with an SAED pattern and the corresponding schematics (**d**). **e** A representative cryo-TEM micrograph of BCCs in water. Inset of **e** is an enlarged image highlighting the BCC wall. **f** WAXD pattern, **g** a DSC first heating thermogram and **h** an SAED pattern of PLLA-*b*-PEG BCCs. Inset of **h** shows a corresponding BCC bright field TEM micrograph. Scale bars in **a**, **b**, **c**, **e**, inset of **e**, **h** are 500, 100, 500, 50, 10, and 200 nm, respectively

Figure 2a, b show scanning electron microscopy (SEM) and transmission electron microscopy (TEM) images of the PLLA-*b*-PEG crystalsome that was formed after emulsion-solution crystallization for 9 days. Spherical capsules are seen in both images with a diameter of ~200 nm, which is consistent with dynamic light scattering (DLS) results (Supplementary Figure 1). Atomic force microscopy (AFM) experiments also confirm the spherical morphology (Supplementary Figure 2). In contrast, PLLA-*b*-PEG single crystals grown from toluene solution instead of toluene/water interface at 25 °C are flat with a lozenge shape (Fig. 2c), with the PLLA crystalline lamella sandwiched by PEG polymer brushes on both sides. (Fig. 2d)^14^. The observed spherical capsules in Fig. 2a also appear partially collapsed, which might be due to the sample drying/deposition process. Cryo-TEM imaging of the crystalsome in aqueous solution was then performed to examine the intact morphology of the capsules. As shown in Fig. 2e, the original shape of the PLLA-*b*-PEG crystalsome in solution is nearly spherical. Similar electron density in the interior of the capsule (area I) and background (area II) suggests that the crystalsome is hollow. To further prove BCCs are hollow and verify the shell thickness, AFM measurement was conducted on the small crystal pieces after breaking the BCCs by ultrasonication (see later discussion). The spherical morphology is similar to our previously reported homopolymer PLLA crystalsomes^19^. Since PLLA-*b*-PEG is used in the present case, we coin the name BCCs to highlight the copolymer nature of the structure. The morphology and size of the BCCs resemble those of the spherical polymersomes. Figure 2e also reveals that, unlike most reported polymersomes, these BCCs exhibit relatively rough contour, which is due to polymer crystallization. An enlarged cryo-TEM image of the red box area shows the shell thickness is approximately 2–3 nm, which is attributed to the crystalline PLLA layer. PEG layer is solvated in aqueous solution that constitutes the lighter layer on the exterior of the BCCs of ~12 nm, as the blue arrow pointed. The thickness of the PLLA crystalline shell (2–3 nm) is similar to that of liposomes and much thinner than the hydrophobic core thickness of PLA-*b*-PEG (*M*_*n*_ = 3.2k−2.8 kg mol^−1^) polymersomes (e.g. ~10.4 ± 1.4 nm)^22^.

To understand the crystalline structure of PLLA, wide angle X-ray diffraction (WAXD) experiments were conducted on dried BCC samples at 25 °C (Fig. 2f). The WAXD pattern concludes that orthorhombic α form of PLLA is formed^19^. The strongest diffraction peak at 16.7° is from (110)/(200) planes of PLLA crystals while the peak at 19.8° is from (203) plane of PLLA and (120) plane of PEG, and the peak at 22°–24° can be attributed to (032) plane of PEG and (210) plane of PLLA^19,23^. Note that the PEG crystals are formed during BCCs solution drying process. Figure 2g shows a differential scanning calorimetry (DSC) thermogram of the BCCs, where a heating rate of 10 °C min^−1^ was used. The two peaks in the thermogram can be attributed to the melting of PEG (at 49.0 °C) and PLLA (at 138.9 °C) crystals, respectively. By integrating the PLLA melting peak and using 91 J g^−1^ as the melting enthalpy value for 100% PLLA α form crystal, the crystallinity of PLLA can be estimated to be 67%^24^. To further exam the PLLA chain orientation in BCC, selected area electron diffraction (SAED) patterns on individual PLLA-*b*-PEG BCCs were recorded, and the results are shown in Fig. 2h. PLLA (200) and (110) diffraction spots are observed, suggesting the *c*-axis and the polymer chains are along the radial direction of the crystalsome. The diffraction spots are arc-shaped, which is attributed to the continuous lattice splay/distortion in order for the single crystal to fit into the curved nano-sized space^19,25^. The SAED pattern therefore confirms the crystalsome shell is made of single crystal-like lamellae with polymer chains normal to the interface.

**Fig. 3:**
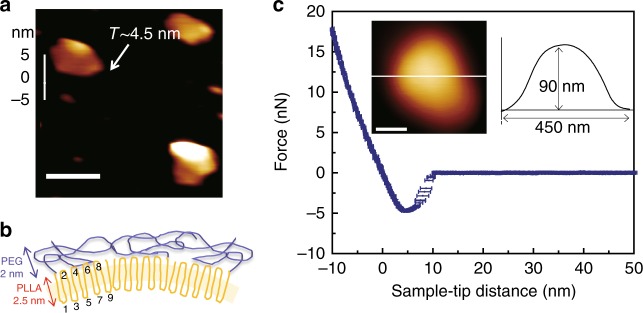
Mechanical properties of PLLA-*b*-PEG BCCs. **a** AFM image and corresponding height profile of a small crystal piece broken using ultrasonication; **b** Side-view of PLLA-*b*-PEG BCC shell in dried state, showing thickness of individual layer and PLLA chain folding; **c** AFM force-deformation spectrum on a PLLA-*b*-PEG crystalsome. Error bars represent standard deviation of sample-tip distances at different forces, *n* = 14. Inset: AFM image of the BCC used for the indentation test and its height profile. Scale bars are 100 nm

To further verify the shell thickness, AFM measurement was conducted on the small crystal pieces after breaking the BCCs by ultrasonication. AFM image and the corresponding cross-sectional height profile (Fig. 3a) shows a total thickness of the shell is ~4.5 nm, including both PEG and PLLA layers. This is significantly thinner than the flat PLLA-*b*-PEG single crystals (11.1 ± 0.2 nm (*n* = 10), supplementary Figure 3). Based on the density and molecular weight of each segment, the thickness of PEG and PLLA layer can be estimated to be 2 and 2.5 nm respectively, which is consistent with the cryo-TEM image in Fig. 2c. As the polymer chain folds normal to the crystal surface, and using the molecular weight and the two-chain orthorhombic unit cell of PLLA α form (*a* = 1.066 nm, *b* = 0.616 nm, and *c* = 2.888 nm, Supplementary Note 1) with 10_3_ helical conformation, it can be calculated that each PLLA chain folds nine times, shown in Fig. 3b. The grafting density σ of PEG on the surface can therefore be calculated to be 0.3 PEG chain nm^−2^. Of interest is that PLLA crystallized into such thin lamellae, which leads to the nine-time fold of the polymer chain. In general polymer crystallization, the crystal thickness is determined by the surface free energy of the crystal folded surface, the heat of fusion, and undercooling of the crystallization process^26–28^. The present case is more complex. In addition to the above-mentioned parameters, entropy loss of the PEG brush upon PLLA crystallization, the molecular weights of the polymer, and the droplet size should all factor in determining the final thickness of the PLLA crystals.

On the basis of PEG chain grafting density, the distance between the anchoring points of the adjacent PEG chains, *D*, is 1.81 nm (See Supplementary Note 1 for calculation of chain folding number, grafting density and adjacent PEG distance). Flory radius of 5000 g mol^−1^ PEG in water can be calculated following *R*_*F*_≈*N*^3/5^*a*^29^, where *a*, *N* and *R*_*F*_ are defined as monomer size, the degree of polymerization and Flory radius. In the present case, *a* = 0.35 nm and *N* *=* 113^30^, and the calculated *R*_*F*_ = 5.95 nm, much greater than the adjacent chain distance 1.81 nm^29^. Therefore, PEG layer in aqueous will take brush conformation, and the brush thickness *L* can be calculated based on $L \approx Na^{({5}/{3})}D^{ - ({2}/{3})}$^29^. Plugging in the above-mentioned values of *N*, *a*, and *D*, the calculated *L* is 13.2 nm, which is consistent with the cryo-TEM observation (Fig. 2c). Note that because the distance between the anchoring points of adjacent PEG is directed by PLLA crystallization, the PEG grafting density can be precisely controlled by PLLA chain folding and the lamellar thickness^31,32^. The spatial distribution of the brush molecules on the surface is extremely uniform due to the crystallization of PLLA, similar to our recently demonstrated polymer brush synthesis using polymer single crystals as the template^33^. This controlled uniform brush layer may improve the circulating performance of the BCCs, which is discussed in the following sections. We also anticipate that the grafting density can be further tuned by changing polymer molecular weights as well as the crystallization condition as previously reported^33^, which will be the focus of our future work.

**Fig. 4:**
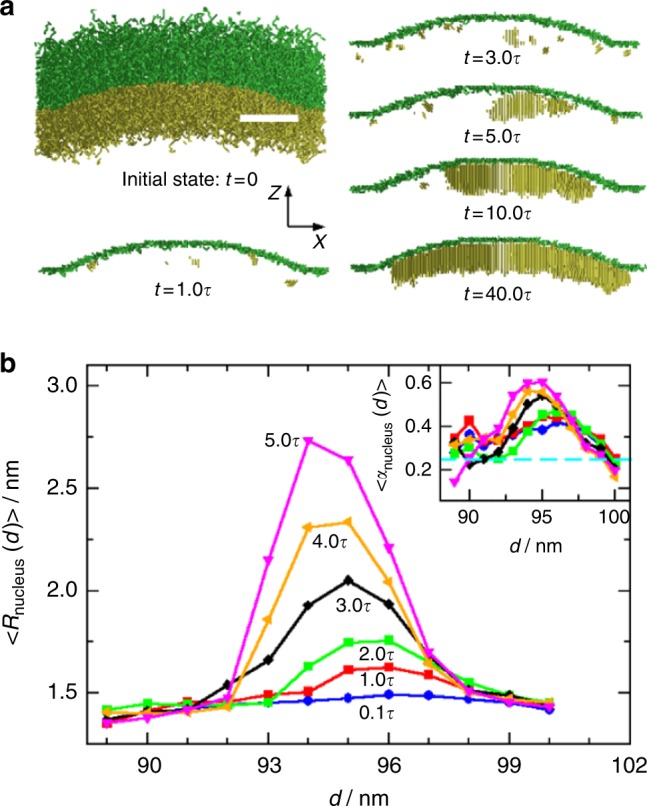
Simulation study of the PLLA crystals grown near the curved liquid−liquid interface. **a** Snapshots of initial state for the PLLA-*b*-PEG chains near the curved interface and the crystallized PLLA bonds at *t* = 1.0*τ*, 3.0*τ*, 5.0*τ*, 10.0*τ*, and 40.0*τ*. The snapshots are plotted in the range of 46 nm < *y* < 56 nm, where *y* = 51 nm is the cross-section *xz* plane through the droplet center. The yellow and green colors show the PLLA and PEG components correspondingly, and the green lines at different *t* show the position of liquid−liquid interface. **b** Radial distributions of average nucleus size at different time *t* = 0.1*τ*, 1.0*τ*, 2.0*τ*, 3.0*τ*, 4.0*τ*, and 5.0*τ*. Here the average is taken from 24 independent simulations, and the positions of nuclei/crystals are determined by its center of mass (CM). Inset shows the radial distribution of average nuclei orientation at different time, with the same color coding as that in the main panel. The dashed cyan line in the inset indicates that the nuclei have no preferred orientation statistically (see Supplementary Note 2). Scale bar in **a** is 20 nm

To understand how the PLLA chains crystallize near the liquid−liquid interface, we used molecular simulations to study the nucleation kinetics (simulation details are in Supplementary Methods)^34,35^. Figure 4a shows the snapshots of the initial PLLA-*b*-PEG chains located at the curved interface, and the PLLA nuclei at different times, *t* = 1.0*τ*, 3.0*τ*, 5.0*τ*, 10.0*τ*, and 40.0*τ* (here *τ* is the longest relaxation time of PLLA chains in solution^36^, Supplementary Figure 5). One can see that at *t* = 1.0τ, there are some small PLLA nuclei formed near the interface. As time increases, these nuclei continue to grow, and some smaller ones dissolve or are merged into the growing nuclei (see the time evolution of average nucleus number and size in Supplementary Figure 6). Eventually only one single crystal is left in the system (see snapshots at *t* = 10.0*τ* and 40.0*τ*).

To identify the accurate position where the nucleus grows, we calculate the radial distribution of the average nucleus size *R*_nucleus_(*d*) (see definitions in Supplementary Note 2), here *R*_nucleus_ is the radius of a virtual spherical nucleus (see Supplementary Note 2) and *d* is the distance between the center of mass (CM) of nucleus and the droplet center. Figure 4b shows the variation *d* of *R*_nucleus_(*d*) at different *t*. One can find that from *t* = 1.0*τ* to 5.0*τ*, the nucleus grows at *d* ≅ 94.5~95.5 nm, rather than from the PLLA-*b*-PEG block junctions at the interface (*d* ≅ 98~100 nm). This slightly off-interface growth can be understood by the radial distribution of average nuclei orientations (see inset of Fig. 4b and definition in Supplementary Note 2). We found that the nuclei are preferred to be vertical to the interface at *d* ≅ 95 nm, while near the interface (*d* ≅ 98~100 nm), they have no preferred orientation. This can be explained as follows: the PLLA chains are stretched vertically to the curved interface due to the high grafting density^37^, giving rise to a preferred nuclei orientation. However, this orientation would be suppressed near the liquid−liquid interface due to the influence of PEG and water solvents. Therefore, in average, the nuclei are grown in the droplet slightly off the liquid−liquid interface. Eventually, the crystal will cover the whole inner surface of the droplet^38^, forming a single crystal-like crystalsome similar to Fig. 2e.

### Mechanical property of PLLA-*b*-PEG BCCs

There are two major methods to characterize the mechanical property of polymer vesicles, namely micropipette aspiration technique and AFM-based nano-indentation^39^. Due to their small size, the mechanical property of PLLA-*b-*PEG BCCs was measured via AFM nano-indentation. In the experiment, aqueous solution of PLLA-*b*-PEG BCCs was drop casted onto a precleaned glass slide and dried under ambient condition. A crystalsome with smooth surface was chosen for the indentation experiment^40,41^. AFM image and the height profile of the crystalsome were acquired under Tapping Mode, as shown in Fig. 3c inset. The force-deformation curve is shown in Fig. 3c, averaged from 14 times of indentation and the error bars represent standard deviation. After indentation, another AFM image was taken (Supplementary Figure 2) to confirm that the original BCC morphology was not deformed, indicating elastic deformation of the BCC during the indentation experiment. The slope of the fitted line of the deformation portion of the curve was used as the shell stiffness *k*_shell_. The Young’s modulus of the dry state PLLA-*b*-PEG shell can be calculated to be 11.5 GPa^42,43^. The membrane bending modulus of the BCC shown in Fig. 3 in dry state can therefore be determined to be 9.8×10^–17^ J (see Supplementary Note 3 for detailed calculation), which is significantly higher than polymersomes or crystalsomes. Note that in aqueous solution, PEG layer would be solvated and will not significantly affect the BCC bending modulus. Therefore the bending modulus of BCCs shell in water can be estimated to be *K*_bend,wet_ = 3.63×10^−17^ J (see Supplementary Note 3 for the calculation). Table 1 summarizes the typical values of sizes and mechanical properties of selected liposomes, polymersomes, homopolymer crystalsomes, BCCs, and viral capsids. It is clear that PLLA-*b*-PEG BCCs have about 140–8000 times higher bending modulus than polymersomes with much greater thickness, even with a much thinner shell thickness. Thus, BCCs shell would be more stable and more robust under shear flow, suggesting they may provide better protection for the potential cargo inside. Interestingly, BCC shares many features with viral capsid: they are spherical, mechanically strong and resilient. The typical thickness of the viral capsid is between 2 and 5 nm, which is also very close to the present BCC structure, suggesting that BCC might provide an ideal self-assembled capsid-mimic system for desired applications such as drug carriers.

**Table 1:** A comparison of mechanical characteristics among liposomes, polymersomes, crystalsomes, and viral capsids.

Self-assembled structures		Diameter (nm)	Thickness (nm)	Young’s modulus (MPa)	Bending Modulus (*k*_B_*T*)
Liposomes	EggPC^59^	37.0 ± 7.9	6 ± 0.6	2 ± 0.8	7
	DPPC^60^	150 ± 20	~5	81	275
Polymersomes	PDMS_68_-*b*-PMOXA_11_^39^	306	16 ± 2	17 ± 11	1703 ± 1216
	PEO_40_-*b*-PEE_37_^47^	2 ~ 5**×**10^4^	8 ± 1	120 ± 20 mN m^−1 a^	30 ± 7
Crystalsomes	PLLA crystalsomes^19^	224	22.5	4160	1.1 × 10^6^
	PLLA-*b*-PEG BCCs	~180	4.5 ± 0.4	(1.1 ± 0.2)×10^4^	(2.4 ± 0.8)×10^5^ (dry)
Viral capsids	Hepatitis B^61^	14.2	2.24	0.26	70.5
	Mature MLV^62^	50	4	1027	1590

EggPC egg yolk phosphatidylchlolin, DPPC dipalmitoylphosphatidylcholine, PDMS-*b*-PMOXA(dimethylsiloxane)-block-poly(2-methyloxazoline), PEO-*b*-PEE Poly(ethylene oxide)-*b*-poly(ethylethylene), MLV murine leukemia virus

^a^Expansion modulus

### In vivo circulation and biodistribution studies of BCCs

**Fig. 5:**
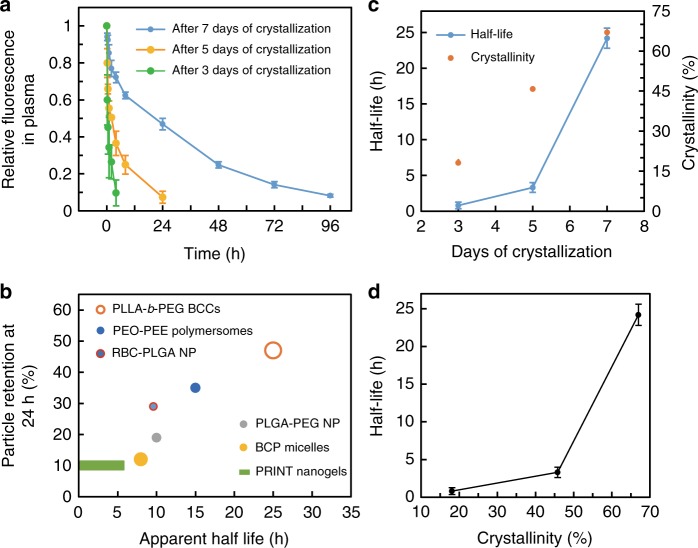
Dependence of BCC blood circulation on their crystallinity. **a** In vivo circulation of DiD-loaded BCCs crystallized for different times. **b** In vivo circulation performance among representative polymeric nanoparticles (PEO-*b*-PEE polymersomes^47^, RBC-PLGA NP^6^, surface-modified PLGA-PEG NP^44^, BCP micelles^56^, and PRINT nanogels^30^) with long blood circulation. A plot of relative intensities at 24 h vs. apparent half-lives, where relative symbol sizes represent relative hydrodynamic sizes/shapes and data are taken from the respective references. **c** BCC crystallinity and half-life variation with different crystallization times. **d** The correlation of circulation half-life and crystallinity of BCCs. Error bars in **a**, **c** represent mean ± s.d, *n* = 3–5

To test the feasibility of using BCCs for intravenous delivery, in vivo circulation experiments were carried out. A hydrophobic fluorescent dye, 1,1′-dioctadecyl-3,3,3′,3′-tetramethylindodicarbocyanine, 4-chlorobenzenesulfonate salt (DiD) was encapsulated in these BCCs during the crystal growth. DiD has been cited as a marker in a number of blood circulation studies for polymeric nanoparticles^6,44^. An in vitro control experiment was firstly conducted to confirm if BCCs are leaky. To this end, DiD-encapsulated PLLA-*b*-PEG BCCs were dispersed in a PBS solution supplemented with 10% fetal bovine serum (FBS). At different time intervals, BCCs were collected, and fluorescent intensity was measured. It was shown that there was minimal fluorescence signal decay (2%) after 5 days of incubation (Supplementary Discussion, Supplementary Figure 8), indicating BCCs are well sealed and nearly impermeable for DiD in this experimental condition. The sealed structure was further demonstrated by the slow release of hydrophilic nitrobenzoxadiazole (NBD)-based dye (~1.7% per 24 h, Supplementary Figure 9). In a separate experiment, BCCs also showed negligible intake of hydrophilic 5-Carboxyfluorescein after 24 h incubation (Supplementary Figure 10). In our in vivo experiments, DiD-encapsulated BCCs with an average size of 200 nm in saline were systemically administered into BALB/c mice through tail vein injection at a dose of 1 mg per mouse. A series of blood samples were collected at various time points post injection. The circulation profiles were obtained by measuring the fluorescence intensity of the sample plasma (Fig. 5a). The blue curve shows the circulation performance of the BCCs crystallized for 7 days. The fluorescence intensity decay follows a classical one-compartment PK model, yielding a long circulation half-life of 24.2 ± 1.4 h (mean ± s.d., *n* = 5). Remarkably, there are 47, 14 and 8% of BCCs remained in the blood after 24, 72 and 96 h circulation, respectively.

Most polymeric nanoparticles administered intravenously are quickly cleared by the mononuclear phagocyte system^45,46^. Numerous strategies have been reported to improve nanoparticle blood circulation time. Nanoparticle size, surface PEGylation and mechanical properties have been shown to affect nanoparticle circulation^45,46^. Figure 5b summarizes the blood circulation apparent half-life and particle retention at 24 h for several reported nanoparticle systems with long circulation lives. Here the apparent half-life is defined as the time for the nanoparticle concentration to fall to half of the original value, and the percentage particle retention at 24 h denotes the fraction of particles retained in plasma 24 h post injection. For example, the reported spherical Poly(ethylene oxide)-*b*-poly(ethylethylene) (PEO-*b*-PEE) polymersome has an apparent half-life 15 h with a 24 h retention of ~35%^47^. Perry et al. reported that with a relatively low PEG brush grafting density, soft hydrogel PRINT nanoparticles showed a half-life 3 h and a 24 h retention of 10%^30^. Hu et al. coated RBC membranes onto PLGA nanoparticles to bypass in vivo clearance, reaching a 9.6 h apparent half-life with 29% retention at 24 h^6^.

Compared to the above-mentioned leading polymeric nanoparticle carriers, BCCs show an impressive half-life of 24.2 h and a 24 h retention of 47%. Furthermore, most nanoparticles investigated in these previous studies are rather small with an average size of 90–100 nm (except for PRINT nanoparticle, which has a dimension of 80 × 80 × 320 nm). It is known that particle size is a key factor for long circulation. Larger nanoparticles of 171 and 243 nm were reported to get cleared in blood twice as fast as that of smaller nanoparticles (80 nm)^46,48^. Given the relative large size of the BCCs, it is even more intriguing to observe the long circulating time value of the present system. Furthermore, previous studies showed that polymer crystallization reduced blood circulation of polymersomes and cylindrical polymer brushes, which is apparently not the case in BCCs^15,49^. To better understand the mechanism of the observed long circulation time of BCCs, we investigated the circulation behaviors of BCCs that were crystallized for shorter periods of time, e.g. 3 days and 5 days. The results are also plotted in Fig. 5a, which shows that BCCs of shorter crystallization time (3-day and 5-day) exhibit much faster clearance in the blood yet 5-day crystallization time yields a blood retention better than 3-day crystallization. Fitted with the one-compartment model, BCCs after 3-day and 5-day crystallizations have half-lives of 0.80 ± 0.46 h (*n* = 4) and 3.3 ± 0.68 h (*n* = 3), respectively. Figure 5c summarizes the change of half-lives with increasing crystallization time. DLS measurement (Supplementary Figure 11a) confirmed that all the BCCs have similar hydrodynamic radii and size distribution. Supplementary Figure 11c, d demonstrate that morphologies of 3-day and 5-day crystallized BCCs also maintained capsule-like structures with similar particle sizes. Interestingly, crystallinity calculated from DSC heating thermograms (Supplementary Figure 11b) illustrated shorter crystallization time rendering lower crystallinity (18.1, 45.8 and 67% for 3, 5, and 7 days of crystallization, respectively), which is also plotted in Fig. 5c. Combining all these results, we can conclude that there is a close correlation between the BCC crystallinity and the blood circulation time, shown in Fig. 5d. The ultra-long circulation time of the 7-day crystallized BCCs arises from the high crystallinity and uniform crystalline chain packing on the curved BCC shells which minimize defect formation in the crystals. In 3-day, 5-day crystallized BCCs and the previously reported polymersome cases, relatively poorly controlled crystals lead to defect-rich particles, which may be prone to disassembly under blood flow. Furthermore, the long circulation may also be facilitated by the uniform PEG brushes formed by crystallization-induced brush-formation. As we recently reported, BCP crystallization-templated polymer brushes are highly tunable and extremely uniform polymer brushes with minimal defects. However, in reported nanoparticle circulation studies, PEG layers are typically formed by grafting-to methods, and are not uniform due to the steric hindrance of the macromolecular chemistry^33^.

**Fig. 6:**
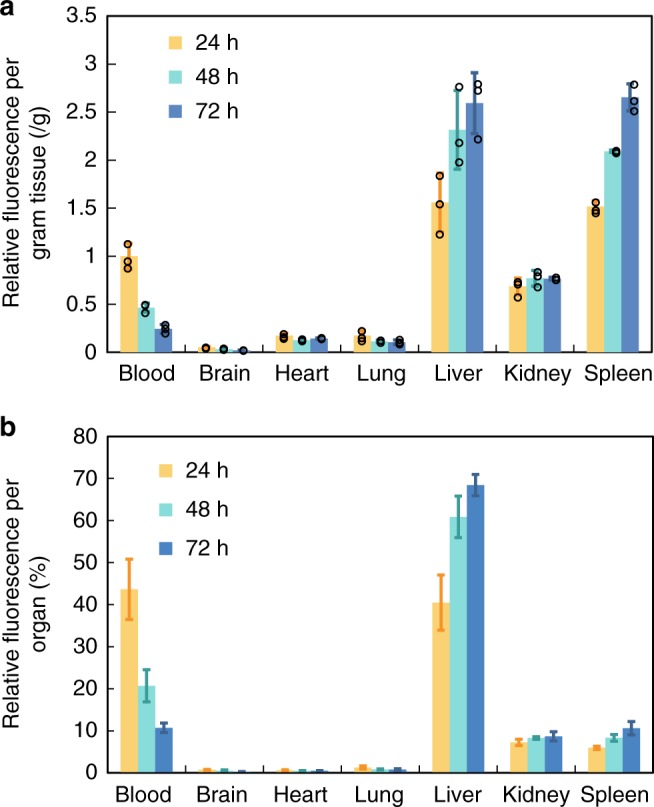
Biodistribution in mice of BCCs after 7 days of crystallization. At different time points (24, 48, and 72 h), fluorescence intensity of homogenized tissues were measured. **a** Fluorescence intensity per gram of tissue. Individual data points are shown in circles. **b** Relative fluorescence signal per organ. Error bars, mean ± s.d, *n* = 3

Nanoparticle distribution in organs was investigated for up to 3 days via IVIS imaging and homogenization (Fig. 6a, b). The relative numbers of PLLA-*b*-PEG BCCs in the blood over 3 days were consistent with the circulation data presented in Fig. 5a that the amount of BCCs in the blood was halved every day, which follows the one-compartment model formula ${{C}}\left( {{t}} \right) = {{C}}({\boldsymbol{t}}_0) \cdot {{e}}^{ - {{k}} \cdot {{t}}_{1/2}}$^50^. With respect to distributions in other organs, the BCCs primarily accumulated to liver and spleen, which are organs of the reticuloendothelial system (RES). Thus, BCCs were mainly sequestered by the liver in such a time frame, which is consistent with the recent understanding on how hard nanomaterials are cleared by the liver^51^. The PLLA-*b*-PEG BCCs accumulation in the liver and spleen increased with time, suggesting that the blood fluorescence mainly arises from the dyes in BCCs rather than the leakage of the dye. In the latter case, the dye would be secreted by kidneys, leading to the decrease of fluorescence signal from the liver^6^.

Polymeric vesicles have been shown to accumulate in the liver^47^. The uptake of nanoparticles by hepatic macrophages, Kupffer cells may be reduced when the liver-specific opsonin absorption is minimal^52^. Besides liver, the PLLA-*b*-PEG BCCs exhibited a high accumulation in the spleen up to 72 h. Feijen et al. reported that polymersomes with a size of 95 nm primarily accumulated in the liver whereas maintained a very low uptake amount in the spleen from 24 to 72 h^53^. PLLA-*b*-PEG BCCs interestingly showed a primary and increasing accumulation over time in the spleen probably due to their relatively larger sizes. It was reported that nanoparticles with size beyond 150 nm are more likely to get entrapped within the liver and spleen^54^. Large stealth liposomes (>200 nm) also showed a significant accumulation in the spleen due to the mechanical filtration in addition to phagocytosis^55^. Application of nanocarriers has long been restricted by the available choices of particle size, while the optimal size was considered to be 70–150 nm^54,56^. PLLA-*b*-PEG BCCs showed that large particles with the size of ~200 nm circulated up to 96 h in the blood, which opens the door for medical applications of large nanoparticles (~200 nm). Furthermore, the hollow structure is advantageous for potential delivery applications since (1) it provides space to encapsulate a large number of drugs at a level that is difficult to achieve by other drug carriers; (2) minimal amount of polymer materials is used in a capsule; and (3) these hollow capsules also provide large surface area to mass ratio. Future research will be focused on better controlling the structure, morphology, size distribution and surface function of the BCCs for targeted applications.

## Discussion

In conclusion, we demonstrated the preparation of robust BCCs for long blood circulation. The emulsion-solution crystallization method is efficient to guide polymer chain folding into uniform crystalline packing at curved liquid/liquid interface to form single crystal-like structure. As a BCP ensemble, BCCs exhibit superior mechanical stability, suggesting that tailoring crystallization is a route to prepare robust BCP vesicles. In vivo circulation tests demonstrate that the BCCs can circulate up to 96 h in the blood with a half-life of 24.2 ± 1.4 h, and 47% retention at 24 h post injection. This excellent circulation performance was attributed to controlled crystallization of PLLA and the resultant uniform PEG brush layer on the BCC surfaces. Our observation is different to the previous perception that crystallization reduces polymersome circulation, providing a strategy to generate long circulating nanomaterials. We envisage that the hollow, robust, tunable BCCs could lead to a new class of nanoparticle carriers for drug delivery and gene therapy.

## Methods

### Materials and animals

Poly (l-lactide)-*block*-poly (ethylene glycol) (PLLA-*b*-PEG, *M*_*n*_ = 6000–5000 g mol^−1^) was purchased from Polymer Source Inc. 1,1-dioctadecyl-3,3,3,3-tetramethylindocarbocy amine perchlorate (DiD oil) was purchased from Life Technologies. Toluene and NaCl were purchased from Sigma Aldrich. All materials were used as received. Female BALB/c mice were purchased from Charles River Laboratory.

### Preparation of PLLA-*b*-PEG block copolymer crystalsomes (BCCs)

22 mg PLLA-*b*-PEG and 12 µg of fluorescent dye (DiD) were first dissolved in 0.4 g toluene in a glass tube. Then, 16 g DI water was added into the above polymer solution at 95 °C. After emulsification at 95 °C for 2 min via probe sonication, the emulsion was kept at 95 °C for another 30 s before quenched to 25 °C for crystallization. After crystallization for 3–9 days, the solution was dialyzed against DI water through 50 nm membranes at 25 °C for 4–6 h, and the procedure was repeated for five times before the sample was collected through a 0.45 µm syringe filter.

Before in vivo study, the above-mentioned BCCs aqueous solution was further dialyzed again saline (0.9 w/v% NaCl aqueous solution) overnight and then concentrated with 30 K molecular weight cutoff (MWCO) Amicon Ultra-4 Centrifugal filters. Gas chromatography experiments showed no detectable amount of toluene in the BCC samples (See Supplementary Methods, Supplementary Figures 12, 13).

### Characterization of PLLA-*b*-PEG BCCs

Hydrodynamic radius were measured by DLS using Zetasizer Nano ZS90. SEM images were taken on a ZEISS Supra 50VP microscope with a 1 kV accelerating voltage. To prepare SEM samples, a drop of 10 µl BCCs aqueous solution was cast onto cover slides and then dried under vacuum overnight. Before SEM imaging, the sample was coated with Pt/Pd.

TEM bright field imaging and SAED experiments were conducted using a JEOL JEM2100 microscope with a 120 kV accelerating voltage. TEM samples were prepared by drop casting BCCs’ aqueous solution onto a carbon-coated Cu grid. Cryogenic-transmission electron microscopy (cryo-TEM) was performed on a FEI Talos F200C TEM (University of Delaware, DE). A vitrified sample was prepared with an FEI Vitrobot apparatus^57,58^. The vitrified samples were transferred to a cryoholder in a sample stage immersed in liquid nitrogen. The cryoholder was kept below –170 °C during the imaging to prevent sublimation. The digital images were recorded by a Talos F200C TEM equipped with a FEI Falcon direct electron detector.

AFM images and force spectra were acquired using a Bruker Dimension Icon AFM equipped with a tapping mode and PeakForce mode. Samples were dried on piranha-cleaned cover slide. The cantilever used is Bruker TESPA with tip height of 10–15 μm, and tip radius of 8 nm. The spring constant of the cantilever is 2.030 N m^−^^1^, calibrated by the thermal tune method. DSC experiments were conducted on DSC Q2000 with Tzero pans from TA Instruments with a 10 °C min^−^^1^ heating rate.

### Monte Carlo simulation

We use a coarse-grained lattice model to study the nucleation kinetics of PLLA chains near the curved liquid−liquid interface. The PLLA-*b*-PEG chain is modeled as a diblock copolymer of A_38_ B_28_, with A and B corresponding to the PEG and PLLA monomers, and the total monomers in one chain is *N* = 66. The curved liquid−liquid interface is set up in prior in the simulation box of 100 × 100 × 99 nm^3^, then PLLA-*b*-PEG chains are relaxed in this interface. The total chain number is chosen as *n* = 3000, calculated from the experimental grafting density 0.3 chains per nm^2^. The polymer chains are moved via a micro-relaxation model^34^, which allows each segment to change positions with its neighboring solvent sites, accompanied by the sliding diffusion along the chain direction if necessary^35^. Conventional metropolis sampling was employed in each micro-relaxation step. The reduced temperature is set as *k*_B_*T*/*E*_*c*_ = 3.6, and other parameters are summarized in Supplementary Note 2. Information.

### In vitro release study

PLLA-*b*-PEG BCCs labeled with DiD was dispersed in a 10% FBS PBS solution. The mixture was shaken at 100  rpm at 37  °C. At each time interval, BCCs were collected with an MWCO 100  K (Amicon Ultra-4) tube with centrifugation. Equal volume of 10% FBS in PBS was used to resuspend BCCs. The fluorescent intensity was measured at an excitation/emission wavelength of 600/665  nm by a microplate reader (Infinite M200, TECAN) and compared with the initial intensity for quantification.

### In vivo circulation study

PLLA-*b*-PEG BCCs labeled with DiD in saline was systemically administered through tail vein injection. Each female BALB/c mouse was slowly injected with 100 μL 10 mg mL^−1^ of NP solution. A small volume of 10 µL of blood was collected at 2 min, 15 min, 0.5 h, 1 h, 2h , 4 h, 8 h, 24 h, 48 h and up to 96 h post i.v. injection. The blood was diluted in 200 μL of heparin/PBS (16 U mL^−^^1^) solution to avoid blood coagulation. The samples were centrifuged at 300 × *g* for 5 min to remove blood cells, and then 180 µL of the supernatant was collected for measurement at excitation/emission wavelength of 600/665 nm by a microplate reader (Infinite M200, TECAN). One-compartment pharmacokinetics model was used to calculate in vivo circulation half-lives via PKSolver^49^. All animal procedures were conducted according to the protocols of the Committee on Animal Care of Drexel in compliance with NIH guidelines.

### In vivo biodistribution study

Female Balb/c mice at 8 weeks old were randomly separated into three groups and were i.v. injected with 150 μL of DiD-labeled PLLA-*b*-PEG BCCs at 10 mg mL^−^^1^ on days 1, 2, and 3 respectively. The control group was injected with the same amount of saline. On day 4, all the mice were sacrificed and their brains, lungs, hearts, livers, spleens, kidneys as well as blood were collected. To quantify the amount of PLLA-*b*-PEG BCCs in each organ, all the organs were weighted and homogenized. The fluorescence intensities of DiD-containing homogenized solution were detected at an excitation/emission wavelength of 600/665 nm by a microplate reader (Infinite M200, TECAN).

### Data availability

The data are available from the corresponding author upon reasonable request.

### Code availability

The simulation code is available from the corresponding author upon request.

## Electronic supplementary material

Supplementary Information
