# Supplementary material for: Block copolymer crystalsomes with an ultrathin shell to extend blood circulation time
Source: Nat Commun. 2018 Aug 1;9:3005. doi: 10.1038/s41467-018-05396-x (PMC6070537; doi:10.1038/s41467-018-05396-x)
Supplement: Supplementary file 1 — Supplementary Information [file 41467_2018_5396_MOESM1_ESM.pdf]

Supplementary Information

for

Block copolymer crystalsomes with ultrathin shell to extend blood circulation time

Hao Qi[a,1], Hao Zhou[a,1], Qiyun Tang[b], Jee Young Lee[c], Zhiyuan Fan[a], Seyong Kim[a], Mark C. Staub[a], Tian Zhou[a], Shan Mei[a], Lin Han[d], Darrin J. Pochan[c], Hao Cheng [a,2], Wenbing Hu[e], Christopher Y. Li[a,2]

[a]*Department of Materials Science and Engineering, Drexel University, Philadelphia, Pennsylvania 19104, United States*

[b]*Institut für Theoretische Physik, Universität Göttingen, Friedrich-Hund-Platz 1, 37077 Göttingen, Germany*

[c]*Department of Materials Science and Engineering, University of Delaware, Newark, Delaware 19716, United States*

[d]*School of Biomedical Engineering, Science & Health Systems, Drexel University, Philadelphia, Pennsylvania 19104, USA*

[e]*Department of Polymer Science and Engineering, State Key Lab of Coordination Chemistry, School of Chemistry and Chemical Engineering, Nanjing University, 210023 Nanjing, China*

[1]H. Q and H. Z contributed equally to this work.

[2]Corresponding Author: chrisli@drexel.edu; hcheng@drexel.edu

Keywords: polymer self-assembly, polymer crystallization, crystalsome, nanoparticle blood circulation.

Supplementary Methods.

Coarse-grained model of PLLA-b-PEG at the curved interface

Experimentally, the molecular weight (M_w) of PLLA-*b*-PEG corresponds to 6000-5000 g mol^{-1}. The repeat unit of PEG is -[O-CH_2-CH_2]$_n$-, with a M_w of $44\ g\ mol^{-1}$. This gives rise to the monomer size of $0.35\ nm$ and the degree of polymerization (*DP*) of 113. Similarly, the PLLA has repeat unit of -[O-CH(CH_3)-CO]$_n$-, with the $M_w = 72\ g\ mol^{-1}$ and *DP*=83. The monomer size of PLLA is similar to that of PEG due to the same number of atoms along the backbone of molecule.

In our simulation, we choose 3 repeat units as a monomer, which generates the monomer size of $1.05\ nm$, closing to that of the Kuhn segment. With this coarse-grained procedure, the experimental PLLA-*b*-PEG is modelled as a diblock copolymer$A_{38}B_{28}$, with A and B correspond to the PEG and PLLA monomers. The total monomer number is $N = 38 + 28 = 66$. Here we ignore the slight volume difference between the PEG and PLLA monomers, and set each monomer size as $b_0 = 1.0\ nm$.

We use the lattice mode [1] to study the crystallization of PLLA-*b*-PEG molecule on the curved liquid-liquid interface. In experiment, the diameter of the formed droplet is around 200 nm. To fully model this droplet and also the phase separation of PLLA-*b*-PEG on the droplet surface, the length of required system box can be estimated as $(200 + 38 \times 2)\ nm = 276nm$, giving rise to a system size of $276 \times 276 \times 276 = 21024576$, about 21 million grid sites. This is too large to be captured by our simulation.

Here we choose a smaller system of $101 \times 101 \times 100$ sites, with the periodic conditions in x, y, and z axis. As each monomer of PLLA-*b*-PEG chain is located on one lattice site, the lattice spacing is naturally equal to the bond length of b_0=1.0 nm. Thus the system size corresponds to the real system of $100 \times 100 \times 99\ nm^3$. We set the center of the droplet sphere at $r_{droplet} = (51, 51, -45)$ (note: this point is not in the simulation box, only used to define the curved interface), and the maximum point along z axis of the curved interface is $A = (51, 51{,}55)$. We define the curved surface as $S(x, y)$, other points on the spherical droplet surface can be calculated according to the $r_{droplet}$, A and also the droplet radius $R_{droplet} = 100nm$. We also choose a plane at $z(x, y) = 44$, where the droplet surface is lower than

the $z(x, y) = 44$, the curved surface is replaced by this plane. This avoids the defects at boundaries in x and y directions when the periodic boundary conditions are employed.

As in our lattice model, the curved plane has a total area of $100 \times 100\ nm^2$ (here it should be noted that the real curved surface always holds a larger surface area. However, in our cubic lattice, the total lattice sites crossing the curved and planar interfaces are the same, thus we still use the planar area to calculate the total number of chains). The grafting density of chain molecules in experiment is $0.3 \text{chains nm}^{-2}$, then we obtain the total chain number on the curved interface as $n = 3000$.

The motion of polymer chains is generated through a micro-relaxation model,[1] which allows each segment to change positions with its neighboring solvent sites, accompanied by the sliding diffusion[2] along the chain direction if necessary. Conventional metropolis sampling was employed in each micro-relaxation step with the potential energy change:

$$\frac{\Delta E}{k_B T} = \frac{b_0 B_0 + b_1 B_1 + b_2 B_2 + b_3 B_3 + b_p E_p + c E_c}{k_B T} = \left(b_0 \frac{B_0}{E_c} + b_1 \frac{B_1}{E_c} + b_2 \frac{B_2}{E_c} + b_3 \frac{B_3}{E_c} + b_p \frac{E_p}{E_c} + c\right) \frac{E_c}{k_B T} \quad (1)$$

Here E_c is the bending energy for two adjacent bonds connected along the chain and c is the total number of non-collinear connected bond pairs along the chain. E_p represents an excess energy between the nonparallel and parallel packing bonds within the nearest neighboring sites. This driving force for polymer crystallization is only valid for PLLA blocks in our model. k_B is the Boltzmann constant and T is the temperature. Here $B_0$and B_3 correspond to the repulsion between PEG-PLLA and toluene-water, and $B_1$and B_2 refers to the repulsion between PEG-toluene and PLLA-water. We consider the interactions between PEG-water and PLLA-toluene as 0, indicating the athermal solvent condition. The factors $b_0,\ b_1,\ b_2,\ b_3,\ b_p$ are the total number of corresponding contact pairs. In our simulation, we choose $B_0/E_c = B_1/E_c = B_2/E_c = 3.0$ to show the strong repulsion between the hydrophobic and hydrophilic components. A higher magnitude of the repulsion between two solvents (toluene-water) is chosen as $B_3/E_c = 5.0$. The driving force of polymer crystallization is set as $E_p/E_c = 1.0$, which is only valid for the PLLA block. The reduced temperature is set as $k_B T/E_c = 3.6$ in the simulation.

In our simulation, these chains are randomly generated on the curved interface, with the PLLA in the inner and PEG at the outer region. Then the chains are relaxed at $k_B T/E_c = 3.6$ for

40000 MCS to obtain the initial state of PLLA-*b*-PEG, see snapshot in Figure 4a in the main text.

Additional BCC Permeability tests

5-Carboxyfluorescein was purchased from Sigma Aldrich. Succinimidyl 6-(N-(7-nitrobenz-2- oxa-1,3-diazol-4-yl)amino)hexanoate (NBD-X, NHS ester, or NBD-X, SE) was purchased from AnaSpec . 1,1-dioctadecyl-3,3,3,3-tetramethylindocarbocy amine perchlorate (DiD oil) was purchased from Life Technologies. All materials were used as received.

We further investigated the release of dye through BCC using a NBD-based system. We chose the organic soluble dye N-hydroxysuccinimide (NHS) ester form NBD-X, SE which can readily hydrolyze to water soluble NBD-X.[3, 4] In our experiment, we prepared NBD-X SE-encapsulated BCCs following our crystallization method. After crystallization for 5-7 days, the BCC solution was washed with Amicon ultra centrifugal filter units (MWCO 100kDa). The encapsulated NBD-dye can be dissolved in water due to hydrolysis, which was confirmed by breaking the BCCs using sonication, and measuring the NBD fluorescence intensity of water after removing broken BCC crystals using centrifugation.

To test NBD dye release from BCCs to water, one ml of the above mentioned BCC solution was incubated in water for 1 day, and then filtered with the Amicon tube. Filtered solution was collected in a vial. Concentrated NBD-containing BCC solution was recovered to 1 ml by adding DI water. This process was repeated for 5 times (one day for each incubation) to study NBD release from BCC to water. At every time point, NBD fluorescent intensity at 535 nm was measured with an excitation at 465 nm and was used to quantify the release of NBD from BCCs. Blank BCC aqueous solution was used as the background signal. After finishing the release experiment, the BCC solution was sonicated by a probe sonicator (Fisher Sonic Dismembrator; 40% amplitude, 5 mins, pulse: 1 s on and 1 s off) to break BCCs. The NBD-X fluorescent intensity of the water after removing broken BCC crystals using centrifugation was used as the 100% control. Three samples were performed for each time point and the average results and standard deviation are reported.

To quantify hydrophilic dye intake, 2mg PLLA-*b*-PEG BCCs were incubated with aqueous solution of a hydrophilic dye 5-carboxyfluorescein at a concentration of 0.5 mg/ml for 24 h. The fluorescence emission spectrum of the BCC solution was then measured with an excitation wavelength of 480 nm, to determine the BCC dye intake.

Gas chromatography measurements.

A Perkin Elmer Clarus 500 Gas Chromatograph/Mass Spectrometer (GC-MS) was utilized with a Phenomenex Zebron ZB-5MSi (5% methyl phenyl silicone, 30 m x 0.25 mm) column. The column oven temperature was held at 70 ^{o}C for 2 min, then increased to 230 ^{o}C at a rate of 20 ^{o}C min^{-1} and held for 2 min. The injector port temperature was set to 250°C. The carrier gas was helium with a flow rate of 1 mL min^{-1}. The ionization energy was set to 70 eV. The mass spectra were recorded from 40 to 100 *m/z* at 0.2s scan intervals. GC-MS conditions were adapted from reference.[5] The total run time was 12 min, with the first 5 minutes being analyzed once the toluene elution time (~2.4 minutes) was determined. To prepare standards and samples**, t**oluene standards were prepared in methanol (HPLC grade) by preparing a 1000 ppm stock standard (57.7 μL toluene in 50 mL methanol). Dilutions were prepared directly into GC vials to obtain standards with toluene concentrations of 0, 10, 50, 100, and 500 ppm. The total volume in the GC vials was 1 mL. The vials were vortexed before analysis to ensure homogenous mixing. The two samples analyzed were the final BCP crystalsome solutions (2 mg mL^{-1} in water) as described with and without the dye added.

Supplementary Figures.

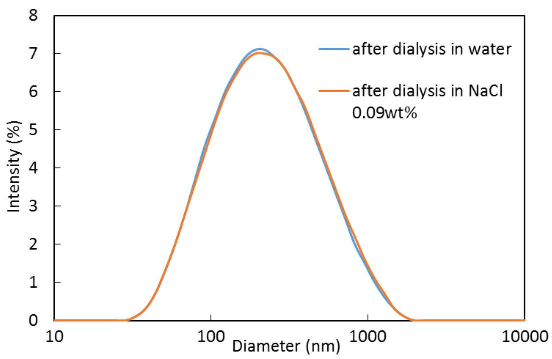

Supplementary Figure 1. PLLA-*b*-PEG BCC size distribution in water and in NaCl 0.09 wt. % aqueous solution as measured by dynamic light scattering (DLS).

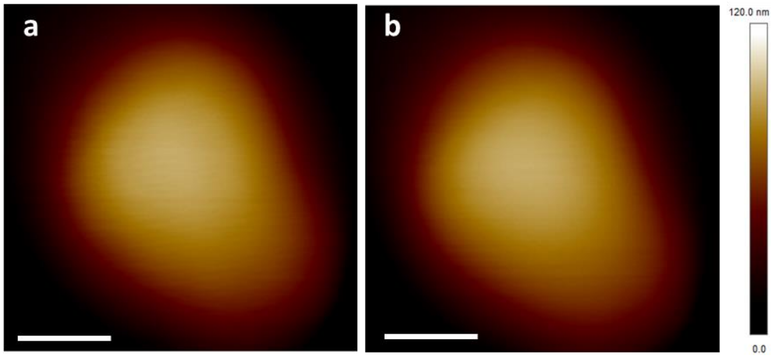

Supplementary Figure 2. AFM height images of PLLA-*b*-PEG BCC. (a) Before and (b) After the nano-indentation test. Scale bars are 100 nm.

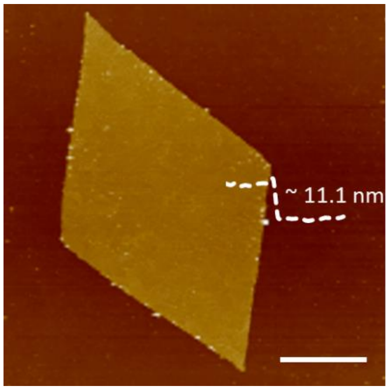

Supplementary Figure 3. An AFM height image with corresponding height profile of a PLLA-*b*-PEG polymer single crystal grown from its 0.05 wt% toluene solution. Scale bar is 2 um. The thickness is 11.1 ± 0.2 nm (mean ± s.d., n=10).

Supplementary Note 1

Calculation of PLLA chain folding number and PEG grafting density

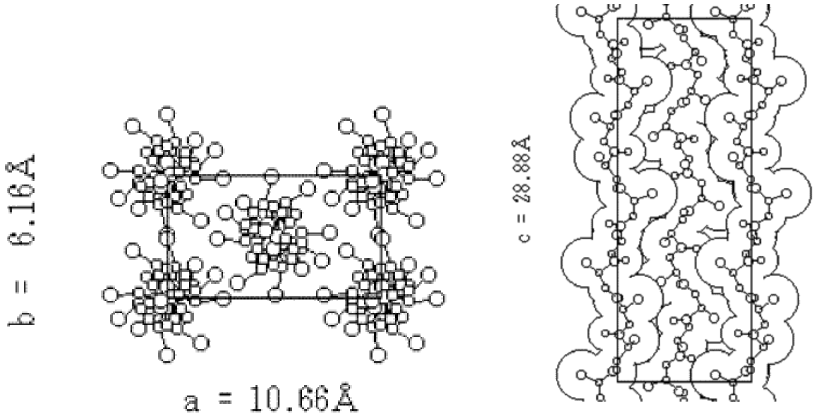

Supplementary Figure 4. *ab* and *ac* projection of crystal structure of 10_3 conformation α-form PLLA.[6] Reprinted with permission from (Sasaki, S. & Asakura, T. Helix distortion and crystal structure of the α-form of poly (l-lactide). Macromolecules 36, 8385-8390, (2003)). Copyright (2003) American Chemical Society.

PLLA segment has a *Mn* = 6000 g mol^{-1} and degree of polymerization (*DP*) = 83. For a 2.5 nm thick PLLA, the folding number can be calculated based on:

$$N = \frac{\frac{DP}{10} \times c}{t} \quad (2)$$

Where *DP* is the degree of polymerization, 10 corresponds to the polymer chain 10_3 helical conformation (Supplementary Figure 4), *c* is the unit cell parameter and *t* is the crystal thickness. Therefore, $N = \frac{\frac{83}{10} \times 2.888}{2.5} - 1 = 9$, indicating each PLLA chain folds 9 times and has 10 stems, indicating that there is a PEG chain every 10 stems of PLLA. According to the *ab* projection of PLLA crystal, there are 2 stems in one unit cell. So each stem occupies an area $S = \frac{1.066 \times 0.616}{2}$ = 0.328 nm^2 and each PEG occupies S_{PEG}=10× S= 3.28 nm^2. Therefore, the PEG neighboring chain distance D can be estimated to be D = $(S_{PEG})^{0.5}$ = 1.81 nm and PEG grafting density $\sigma = \frac{1}{0.328 nm^2 \times 10} = 0.3$ nm^{-2}.

Supplementary Note 2

Monte-Carlo simulation

Time scale

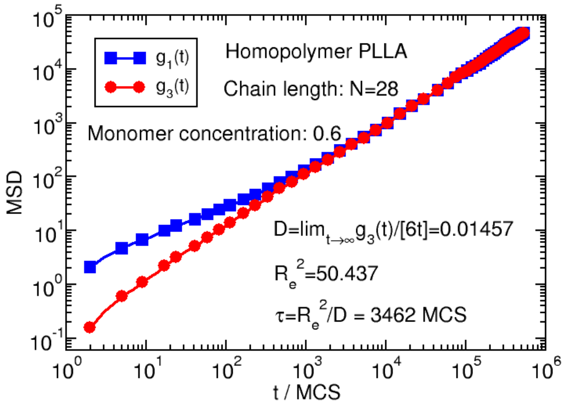

Supplementary Figure 5. Time evolution of mean-square displacements of monomers, $g_1(t)$, and center of mass for polymers, $g_3(t)$ as a function of time *t* (in unit of Monte Carlo steps) at monomer concentration *c*=0.6 and $k_BT/E_c = 3.0$. Inset equations show the self-diffusion coefficient *D*, the square of end-to-end distance $R^2{}_e$, and the longest single-chain relaxation time,τ, obtained from the simulation results.

Here we set the time unit from a controlled simulation of the homopolymer PLLA with chain length $N = 28$ at the monomer concentration $c = 0.6$. The characteristic time of this system is a good indicator for studying the time evolution of nucleation of PLLA nuclei/crystals near the liquid-liquid interface. We choose the system size of $30 \times 30 \times 30$, with the periodic conditions in x, y, and z axis, and the simulation is performed at reduced temperature of $k_BT/E_c = 3.0$. Each monomer sits on one lattice and unoccupied lattices are the athermal solvents. Supplementary Figure 5 shows the mean-square displacements of monomers, $g_1(t)$, and that of polymer's center of mass, $g_3(t)$.[7] We estimate the self-diffusion coefficient, $D = \lim_{t\to\infty} g_3(t)/6t = 0.01457$, in unit of nm^2/MCS'. The square of end-to-end distance is calculated from the equilibrium configurations of all the chains, $R_e^2 = 50.437\, nm^2$. Thus we can obtain the longest single-chain relaxation time, $\tau = R_e^2/D = 3462MCS$. In the following, we choose τ as the time unit for all the discussions.

Time evolution of the average precursor/nucleus/crystal number and size

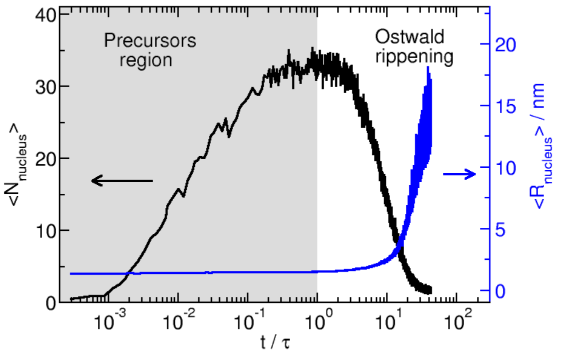

Supplementary Figure 6. Time evolution of the average number $\langle N_{nucleus}\rangle$ (black, left axis) and average radius $\langle R_{nucleus}\rangle$ (blue, right axis) of the precursors/nuclei/crystals. The average is taken from 24 independent simulations.

In this section we study the time evolution of the average number $\langle N_{nucleus}\rangle$ and average size $\langle R_{nucleus}\rangle$ of the crystal nucleus. Here the nucleus is defined as once 3 PLLA bond vectors are parallel to each other in the nearest neighbouring sites. The average is taken from 24 independent simulations. The size of nucleus i can be characterize by the radius of a virtual sphere: $R(r_i) = \sqrt[3]{3N_i b_0^3/4\pi}$, here N_i and $r_i$are the monomer number and center of mass (CM) of nucleus i, and $b_0^3 = 1.0\ nm^3$ is the volume of one monomer. Normally the nucleus is not a perfect sphere, and $R(r_i)$ does not correspond to the real radius of nucleus. However, if we could consider the nucleus as a virtual sphere, then $R(r_i)$ becomes a good quantity to characterize the nucleus growth when a few monomers are adsorbed to the nucleus i. We also use the volume $V(r_i) = N_i b_0^3$ to characterize the nucleus size, the results, such as the average size and its radial distributions, are qualitatively same.

Supplementary Figure 6 shows the time evolution of the average number $\langle N_{nucleus}\rangle$ (black curve, left y axis) and the average radius $\langle R_{nucleus}\rangle$ (blue curve, right y axis) of the formed nucleus. One can find that the average nucleus number experiences difference stages: at the very early stage $0 < t < 1.0\tau$, the number of nucleus increases rapidly, while the average nucleus size increases very slowly. This implies that the nucleus formed in this stage does not grow, which can termed as precursors. Subsequently, the nucleus number decreases during

$1.0\tau < t < 30.0\tau$, while the average nucleus size gradually increases. This behavior is similar to an Ostwald rippening process,[8, 9] during which the small nucleus dissolves and the materials are absorbed by the growing large nucleus. After $t = 30.0\tau$, the average nucleus number reduces to around 1, indicating only one nucleus left in the system. In this stage, the nucleus becomes a single crystal. To simplify the discussion, we use the term “nucleus” to characterize the crystallized clusters during all the 3 stages.

The whole process is a typical nucleation process. To understand whether the nucleus grow from the liquid-liquid interface or from other positions, we study the radial distributions of the average nucleus size, nucleus number, and average orientations in the following sections.

Definition of radial distribution of average nucleus size

We calculate the radial distribution of average nucleus size as:

$$\langle R_{nucleus}(d)\rangle = \frac{\sum_i^{N_{nucleus}} R(r_i)\delta(|r_i - r_{droploet}| - d)}{\sum_i^{N_{nucleus}} \delta(|r_i - r_{droploet}| - d)} \quad (3)$$

Here $r_{droploet}$ is the position of the droplet center, d is the distance between the CM of nucleus i (see next subsection) and droplet center. The radial distributions of $\langle R_{nucleus}(d)\rangle$ at different time t are shown in Figure 4b in the main text.

Radial distributions of the nucleus number

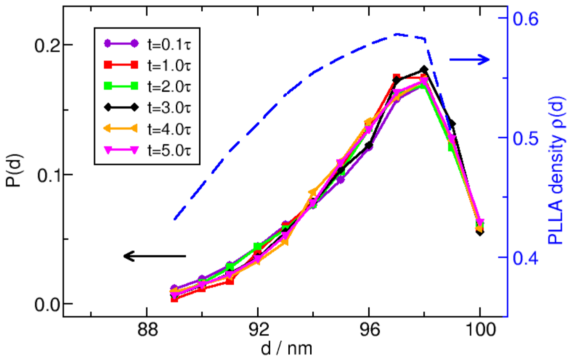

Supplementary Figure 7. Radial distribution of nucleus number at different time $t = 0.1\tau$, 1.0τ, 2.0τ, 3.0τ, 4.0τ and 5.0τ. Dashed blue line indicates the radial PLLA monomer density before crystallization.

Here we calculate the radial distribution of the nucleus number at difference time. The position of nucleus i is defined by its center of mass: $r_i = \sum_i^{N_i} r_j / N_i$, here N_i is the total number of monomers in nucleus i and r_j is the position of monomer j belonging to nucleus i. The radial distribution of nucleus number is calculated by:

$$P(d) = \frac{\sum_i^{N_{nucleus}} \delta(|r_i - r_{droploet}| - d)}{N_{nucleus}} \tag{4}$$

here $N_{nucleus}$ is the total nucleus number in the system. Supplementary Figure 7 shows the radial distribution of $P(d)$ at different time t. As the droplet radius is 100 nm, and the block junctions of PEG-PLLA are located at the droplet surface, thus $d = 100nm$ indicate the interface between PEG and PLLA. One can find that the nuclei are accumulated near the interface and its radial distributions are insensitive to time t. This is mainly attributed to the average radial density of PLLA monomers, see the dashed line in Supplementary Figure 7.

Radial distributions of the average nucleus orientations

To understand why the nuclei do not grow from the interface, we study the radial distribution of average nucleus orientations. To do so, we first need to quantitatively characterize the orientation of one single nucleus i, which can be obtained by defining the following orientation order parameter

$$\alpha(r_i) = \langle\frac{3\cos^2\theta_i - 1}{2}\rangle \qquad (5)$$

Where θ is the angle between the vector of crystallized bonds $\vec{r}_{i+1} - \vec{r}_i$ and that of z axis. If the crystallized bond becomes parallel to the z axis, $\theta = 0$ gives rise to $\alpha(r_i) = 1$. However, if the crystallized bond is vertical to the z axis, $\theta = \pi/2$ and $\alpha(r_i) = -0.5$. In our lattice model, the angle θ between the crystallized nuclei and z axis could be 0, $\pi/4$, $\pi/2$, $3\pi/4$, π, thus the $\alpha(r_i)$ takes the values of -0.5, 0.25, 1.0. Statistically, the nuclei has no preferred orientations means that the probability of $\alpha(r_i) = -0.5$, 0.25, and 1.0 is equal, thus $\langle\alpha(r_i)\rangle = (-0.5 + 0.25 + 1.0)/3 = 0.25$, see the dashed cyan line in inset of Figure 4b in the main text.

The radial distribution of average nucleus orientation can be calculated as:

$$\langle\alpha_{nucleus}(d)\rangle = \frac{\sum_i^{N_{nucleus}} \alpha(r_i)\delta(|r_i - r_{droploet}| - d)}{\sum_i^{N_{nucleus}} \delta(|r_i - r_{droploet}| - d)} \qquad (6)$$

Inset of Figure 4b in the main text shows the radial distribution of $\langle\alpha_{nucleus}(d)\rangle$ at different time t. One can see that near the interface $d \cong 98nm$, the $\langle\alpha_{nucleus}(d)\rangle \cong 0.25$, indicating that the there are no preferred orientations for the nuclei. However, at $d \cong 95\ nm$, the $\langle\alpha_{nucleus}(d)\rangle \cong 0.5$, implying that the nuclei are preferred to be parallel to the z axis. When $d < 95nm$, the $\langle\alpha_{nucleus}(d)\rangle$ decreases again, which can be attributed to the random coil trend of the PLLA chains due to the lower monomer densities (see Supplementary Figure 7).

Supplementary Note 3

Calculation of bending modulus of PLLA-*b*-PEG BCCs.

By linear fitting the deformation portion of the AFM curve (Figure 3c in the main text), the slope of the fitted line corresponds to the shell stiffness k_{shell}.

The equation relating the stiffness k_{shell} with Young's modulus E has been derived in the classical shell theory:[10, 11]

$$k_{shell} = \frac{4Eh^2}{R\sqrt{3(1-\nu^2)}} \qquad (7)$$

Where h is the shell thickness, v is the Poisson ratio and R is the curvature radius of the crystalsome's spherical cap which can be calculated from the AFM measurements of its width W and height H,

$$R = \frac{0.25W^2 + H^2}{2H} \qquad (8)$$

The corresponding membrane-bending modulus K_{bend} can be further calculated:

$$K_{\text{bend}} = \frac{Eh^3}{12(1-\nu^2)} \qquad (9)$$

For the calculation of dry state BCCs, the shell thickness h was measured from broken shell pieces, which is 4.5 nm. A Poisson ratio a value of ν = 0.33 was used,[12] and the curvature radius was calculated to be 325 nm (eq. 8). The calculated stiffness obtained is 1.75 N/m (std. 0.08N/m, n = 14). The Young's modulus of the PLLA-*b*-PEG shell is approximately 11.5 GPa (eq. 7), which represents the materials' crystalline nature in such a bilayer composites structure. The membrane bending modulus of the BCC shown in Supplementary Figure 3 in dry state can therefore be determined to be 9.80×10^{-17} J (eq. 9).

For the calculation of BCC bending modulus in water, the shell thickness h of 2.5 nm was used since PEG layer is solvated. There is also approximately 1% water absorbed by PLLA,[13] however water uptake of PLLA will not significantly change the Young's modulus according to a previously report.[14] Correspondingly, the Young's modulus of PLLA can be estimated using:

$$E_{PLLA}=E_{\text{crystal}} * X\text{c} + E_{\text{amorphous}}*(1-X\text{c}) \qquad (10)$$

Where E_{crystal} the theoretical Young's modulus of pure PLLA crystal along its chain direction, Xc is the crystallinity and $E_{\text{amorphous}}$ is the Young's modulus of amorphous PLLA. Respectively, using 0.67, 36 Gpa and 4 Gpa for X_c, E_{crystal} and $E_{amouphous}$.[15] the E_{PLLA} = 25.4 Gpa is calculated. Moreover, based on equation 3 the bending modulus in water can be estimated to be $K_{\text{bend,wet}} = 3.63\times10^{-17}$ J. Another method of estimation is using the previous obtained dry modulus of PLLA-*b*-PEG shell (11.5 GPa) as the PLLA shell modulus in the aqueous state, leading to $K_{\text{bend,wet}} = 1.64\times10^{-17}$ J. Bending moduli obtained from both methods are consistent with each other.

Supplementary Discussion.

Additional BCC Permeability tests.

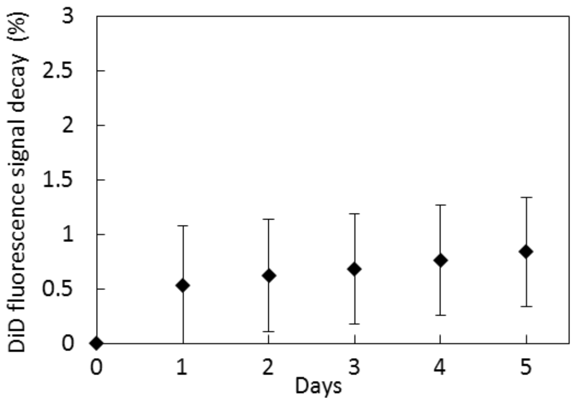

Supplementary Figure 8. DiD release test. *In vitro* fluorescence decay of DiD dye encapsulated in BCCs over 5 days. Error bars represent standard deviation, n=3.

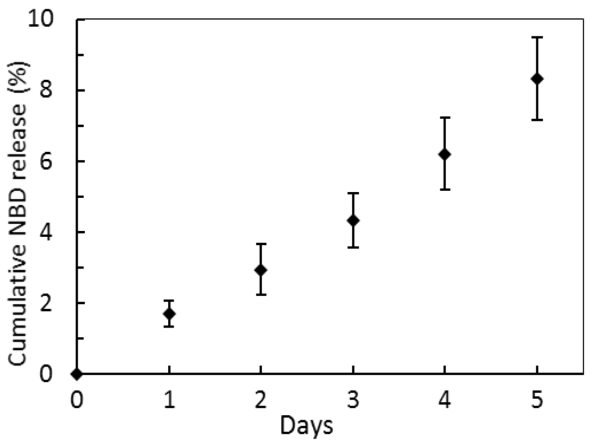

Supplementary Figure 9. Release profile of NBD dye from BCCs. Error bars represent standard deviation, n=3.

Supplementary Figure 9 showed that 1.7 % of NBD was released from the BCCs after 1 day. Each day, similarly small amount of NBD was slowly released. After 5 days, the cumulative release is 8.3 %. Based on this result, we can conclude that the BCCs have relatively low permeability to hydrophilic NBD-X.

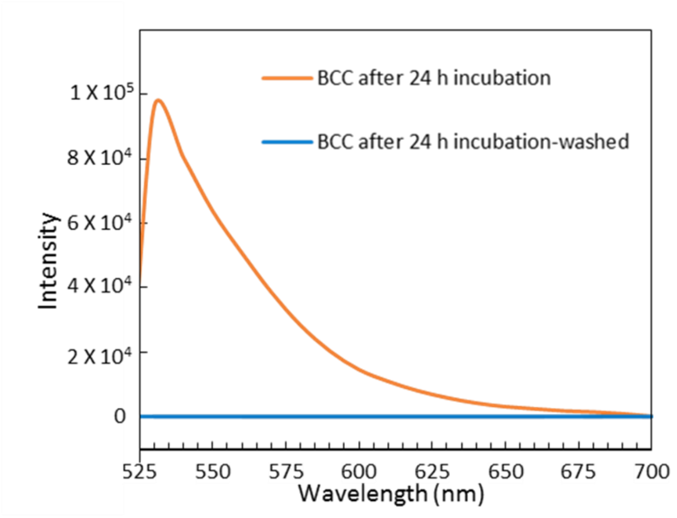

Supplementary Figure 10. Hydrophilic dye intake. Fluorescent emission of BCC after 24 h incubation with 5-carboxyfluorescein and after further wash.

A fluorescent emission peak at ~ 530 nm can be assigned to 5-carboxyfluorescein (Supplementary Figure S10). Subsequently, BCCs were collected using Amicon ultra centrifugal filter units (MWCO 100kDa), and re-suspended in DI water. The BCC sample after incubation/washing showed little fluorescent emission (Supplementary Figure S10, blue line), indicating that the hydrophilic 5-carboxyfluorescein was not able to diffuse into BCC.

Crystallization time effect on BCC structure

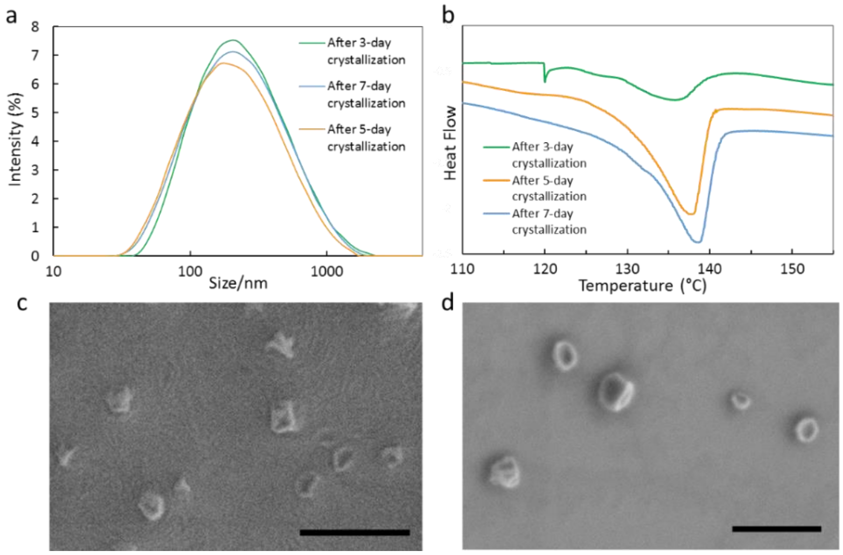

Supplementary Figure 11. (a) PLLA-*b*-PEG crystalsome size distribution in 0.09 wt. % NaCl aqueous solution after different time of crystallization, as measured by DLS. (b) Differential scanning calorimetry thermogram of PLLA-*b*-PEG BCCs of different crystallization times (endotherm down). Scanning electron microscopy images of BCCs (c) after 3 days of crystallization and (d) after 5 days of crystallization. Scale bars in (c) and (d) are 1 um.

Gas chromatography results

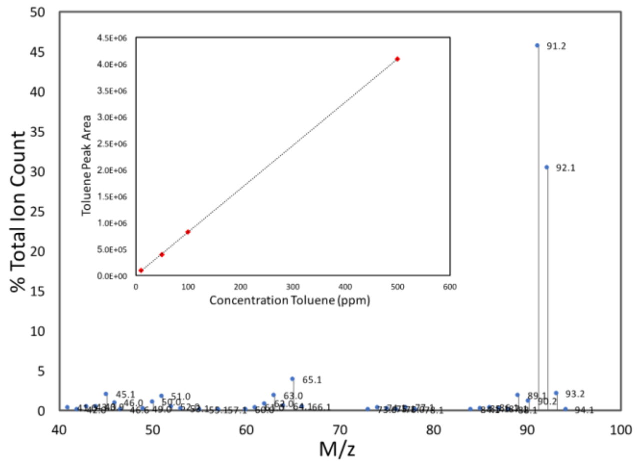

Supplementary Figure 12. Mass spectra recorded during toluene elution interval. Inset shows calibration curve from toluene standard series prepared.

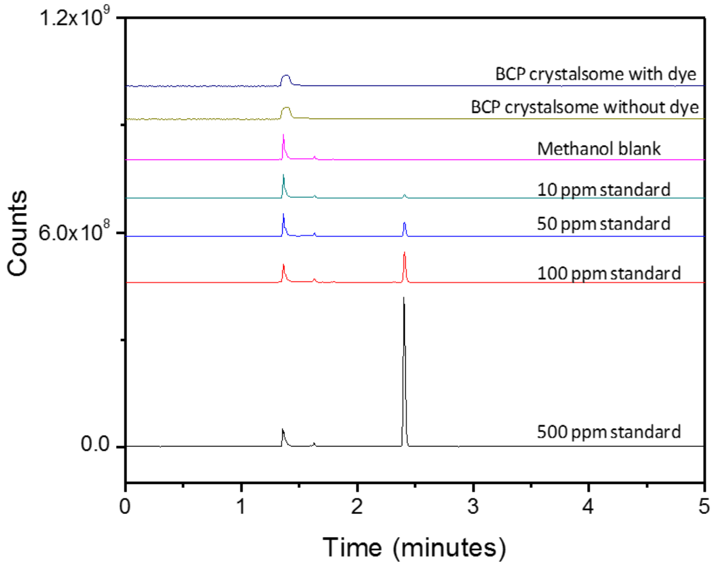

Supplementary Figure 13. Chromatographs of toluene standard series, methanol blank, and BCP crystalsome samples.

Proper column chemistry and GC-MS conditions are confirmed by excellent toluene peak shape, no tailing or smearing. The toluene peak was confirmed by matching the mass spectra recorded during the elution time of the 2.4min peak to toluene spectra from the Perkin Elmer mass spectra library. A representative mass spectra of the 500ppm standard is shown in Supplementary Figure 12. The methanol blank identifies that utilizing this solvent does not influence the toluene peak due to no peak overlap, Supplementary Figure 13. Accurate quantitation of toluene in the samples from 0-500 ppm is achievable with this method as seen by the very good linearity (R^2=0.99997) of the calibration curve, inset Supplementary Figure 12.

The chromatographs, Supplementary Figure 13, show no detectable amount of toluene in either BCP crystalsome sample. Analysis of the mass spectra from the toluene elution interval in the samples showed no detectable toluene mass spectrum. From this it can be concluded that the samples contain no quantifiable amount of toluene that would limit their use *in vivo*.

Supplementary References

1. Hu W, Frenkel D. Polymer crystallization driven by anisotropic interactions. In: *Interphases and Mesophases in Polymer Crystallization III*. Springer (2005).

2. Tang Q, Hu W, Napolitano S. Slowing down of accelerated structural relaxation in ultrathin polymer films. *Phys Rev Lett* **112**, 148306 (2014).

3. Fan Z, Zhou H, Li PY, Speer JE, Cheng H. Structural elucidation of cell membrane-derived nanoparticles using molecular probes. *J Mater Chem B* **2**, 8231-8238 (2014).

4. Ghosh P, Whitehouse MW. 7-chloro-4-nitrobenzo-2-oxa-1, 3-diazole: a new fluorigenic reagent for amino acids and other amines. *Biochem J* **108**, 155 (1968).

5. El-Haj B, Al-Amri A, Hassan M, Bin-Khadem R, Al-Hadi A. A GC-MS method for the detection of toluene and ethylbenzene in volatile substance abuse. *J Anal Toxicol* **24**, 390-394 (2000).

6. Sasaki S, Asakura T. Helix distortion and crystal structure of the α-form of poly (l-lactide). *Macromolecules* **36**, 8385-8390 (2003).

7. Müller M, Daoulas KC. Single-chain dynamics in a homogeneous melt and a lamellar microphase: A comparison between Smart Monte Carlo dynamics, slithering-snake dynamics, and slip-link dynamics. *J Chem Phys* **129**, 164906 (2008).

8. Lifshitz IM, Slyozov VV. The kinetics of precipitation from supersaturated solid solutions. *J Phys Chem Solids* **19**, 35-50 (1961).

9. Wagner C. Theorie der alterung von niederschlägen durch umlösen (Ostwald-reifung). *Zeitschrift für Elektrochemie, Berichte der Bunsengesellschaft für physikalische Chemie* **65**, 581-591 (1961).

10. Reissner E. Stresses and small displacements of shallow spherical shells. I. *J Math Phys* **25**, 80-85 (1946).

11. Reissner E. Stresses and small displacements of shallow spherical shells. II. *J Math Phys* **25**, 279-300 (1946).

12. Greaves GN, Greer A, Lakes R, Rouxel T. Poisson's ratio and modern materials. *Nat Mater* **10**, 823-837 (2011).

13. Lyu SP, Untereker D. Degradability of Polymers for Implantable Biomedical Devices. *Int J Molecul Sci* **10**, 4033-4065 (2009).

14. Orue A, Eceiza A, Pena-Rodriguez C, Arbelaiz A. Water Uptake Behavior and Young Modulus Prediction of Composites Based on Treated Sisal Fibers and Poly(Lactic Acid). *Materials* **9**, (2016).

15. De Oca HM, Ward I. Structure and mechanical properties of poly (L-lactic acid) crystals and fibers. *J Polym Sci, Part B: Polym Phys* **45**, 892-902 (2007).
